# Non-Specific Low Back Pain, Dietary Salt Intake, and Posterior Lumbar Subcutaneous Edema

**DOI:** 10.3390/ijerph19159158

**Published:** 2022-07-27

**Authors:** Ronald B. Brown

**Affiliations:** School of Public Health Sciences, University of Waterloo, Waterloo, ON N2L 3G1, Canada; r26brown@uwaterloo.ca

**Keywords:** non-specific low back pain, edema, dietary salt, sodium chloride, posterior lumbar subcutaneous edema, hypervolemia, stiff joints

## Abstract

Low back pain is the world’s leading disability, but the etiology of the majority of low back pain is non-specific with no known cause. Moreover, overuse of opioids to treat low back pain is a widespread problem. This paper proposes that non-specific low back pain may be associated with excessive intake of dietary salt, potentially mediated by posterior lumbar subcutaneous edema. In addition to pain, symptoms of edema include swelling, tightness, and stiff joints, which are common complaints of people with low back pain, along with restricted lumbar range of motion and impaired mobility. Many global populations consume excess sodium chloride, which can lead to fluid overload in hypervolemia, and cause swelling and temporary weight gain associated with low back pain. Numerous conditions comorbid with low back pain are also potentially mediated by excessive salt intake, including migraine headache, hypertension, cardiovascular disease, venous thromboembolism, liver disease, respiratory disorders, chronic kidney disease, pregnancy complications, and multiple sclerosis. Novel approaches to identify and prevent the cause of non-specific low back pain have potential to reduce disability worldwide by reducing low back pain prevalence. More research is needed to confirm the involvement of dietary salt and posterior lumbar subcutaneous edema in non-specific low back pain.

## 1. Introduction

For the past three decades, low back pain (LBP) has been the world-leading non-fatal cause of disability [1], burdening the U.S. healthcare system with costs exceeded only by diabetes and ischemic heart disease [2]. However, with the exception of LPB arising from pathological mechanisms, specific causes of most LPB (~85%) are unknown [3,4]. Discussing management of non-specific low back pain, the *Lancet* noted that overuse of opioids, diagnostic imaging, and surgery “remains a widespread problem” [5]. Back pain (in general) is the second most common diagnosis for opioid prescriptions in emergency departments, next to non-fracture injuries, and 45.2% of patients with back pain receive opioids [6]. Other researchers pointed out that although opioids have short-term analgesic effects in chronic back pain, “benefits for function are less clear” [7]. Furthermore, “complications of opioid use include addiction and overdose related mortality, which have risen in parallel with prescription rates.” On the other hand, from a primary prevention perspective, the *Lancet* predicted that novel approaches to identify and mitigate the cause of non-specific LBP have “potential to help the many people with disabling low back pain worldwide” [5].

Excess body weight has been associated with overall back pain [8], but not all overweight people have back pain, nor are all people with back pain overweight, implying that other etiological factors are involved. Even highly conditioned athletes commonly have low back pain from a “benign” source [9]. Additionally, low-quality dietary patterns with high intake of sodium, saturated fat, and added sugars have been associated with spinal pain [10]. Excessive sodium in the body can cause increased water retention in edema, which could affect tissues involved in LBP of young and old individuals, regardless of body weight and health status.

This perspective article proposes a biological mechanism in which non-specific LBP is associated with excessive dietary salt intake, potentially mediated by posterior lumbar subcutaneous edema. Using a grounded theory approach to review the research literature [11], evidence based on research findings was retrieved from a keyword search for “low back pain”, “dietary sodium”, “sodium chloride”, “salt”, and “posterior lumbar subcutaneous edema”. Additional keywords were used to search for various conditions comorbid with low back pain. Studies cited in retrieved literature were used to expand the literature search. Research findings were examined by comparative analysis for similarities and differences in concepts, and concepts were combined into interrelated themes. Through an iterative process of retrieving additional information from the literature (theoretical sampling), an evidence-based explanatory theory linking salt with LBP was gradually induced until no more new knowledge was obtained (theoretical saturation). Evidence from the grounded theory presented in this paper provides novel insights and new directions for further research and hypothesis testing in the etiology of non-specific LBP potentially related to salt intake.

## 2. Posterior Lumbar Subcutaneous Edema

**Figure 1:**
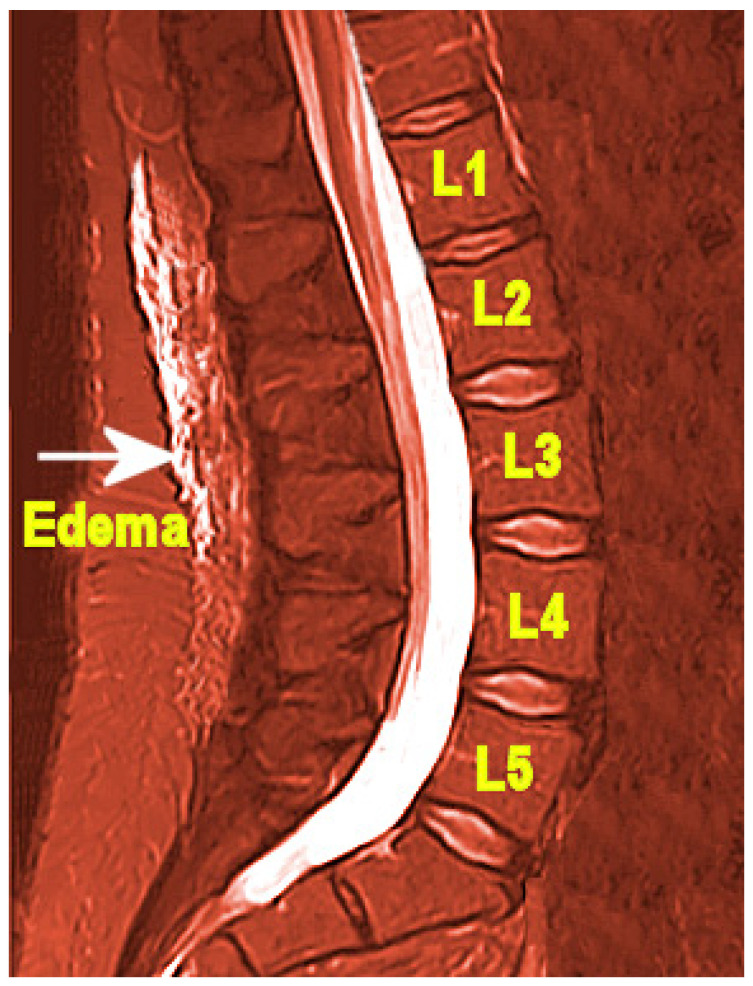
Posterior lumbar subcutaneous edema. Adapted with permission from Schwarz-Nemec et al., 2020 [12].

Posterior lumbar subcutaneous edema (PLSE) affects deep perifascial soft tissue in the lumbar spine (lumbar vertebrae L1–L5), which is commonly ignored as an incidental finding on spinal magnetic resonance images (MRI), shown in Figure 1 [12]. Importantly, PLSE should not be confused with edema caused by serious trauma and injury. In addition to subcutaneous edema, a case control study found that structures within the lumbar spine may also be affected by edema associated with LBP, such as interspinous ligament edema, facet joint effusion, paraspinal muscle edema, as well as neocyst formation [13]. An important study limitation is that “there was no correlation between the lesions and the intensity of symptoms (degree of pain)”.

Symptoms of edema include swelling, tightness, pain, and stiff joints [14], and cases with PLSE may be accompanied by these edema symptoms. Similar to pain and limited mobility with restricted range of motion associated with edema of peripheral limbs [15], LBP is associated with restricted lumbar range of motion and mobility impairment, sometimes in combination with lower extremity pain [16]. Additionally, qualitative studies of patients with LBP report symptoms of stiffness, which is consistent with tightness and stiff joints from edema [17], although the authors noted that “feeling stiff” is not a reliable biomarker of biomechanical impairment. Importantly, the Mayo Foundation for Medical Education and Research, associated with the Mayo Clinic—the top ranked U.S. hospital in 2021–2022 [18], listed adverse effects from medical administration of sodium chloride. Listed symptoms include joint pain, stiffness and swelling when given orally [19] and pain of the low back when injected [20].

## 3. Salt and Hypervolemia

Swelling and temporary weight gain in edema is often associated with fluid overload in hypervolemia due to sodium and water retention; as the body’s total sodium content increases, serum levels of sodium in hypervolemia can be high, normal, or low [21], (hypernatremia, eunatremia, and hyponatremia, respectively). Serum sodium becomes dysregulated when renal function is burdened, especially in combination with high dietary sodium intake [22]. The body’s requirement for sodium is low, 500 mg per day [23]. Yet, salt (sodium chloride) contributes excessive amounts of sodium in the diet of many global populations, with an average salt intake of 9−12 g per day [24]. Importantly, the taste for salt decreases as people are exposed to lower dietary intake levels [25]. Potassium also aids renal elimination of sodium to maintain sodium and fluid balance, but dietary potassium intake is often inadequate as consumption of processed foods increases in many populations.

**Figure 2:**
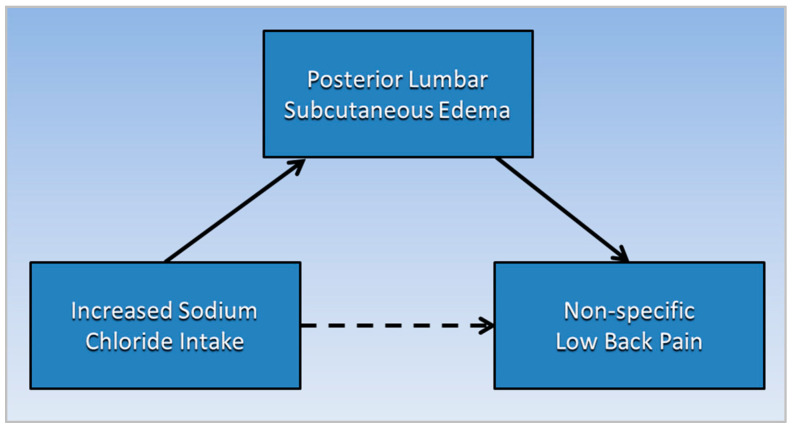
Posterior lumbar subcutaneous edema potentially mediates the association of increased sodium chloride intake with non-specific low back pain.

Figure 2 is a directed acyclic graph illustrating the proposed causative pathways (solid lines) in which posterior lumbar subcutaneous edema potentially mediates the association (dotted line) between increased sodium chloride intake and non-specific low back pain. Note that a mediator, or an intermediate variable, is a causative factor that “lies on the causal pathway” [26].

## 4. Salt Transitively Links Low Back Pain and Comorbid Conditions

Additional evidence that excessive sodium chloride intake is a potential mediator or causative factor in nonspecific LBP is implied in numerous associations of LBP with comorbid conditions mediated by salt, including migraine headache, hypertension, cardiovascular disease, venous thromboembolism, liver disease, respiratory disorders, chronic kidney disease, pregnancy complications, and multiple sclerosis.

Analysis of research findings in this section uses a transitive inference method, inferring that salt is a common causative factor related to both non-specific LBP and comorbid conditions that are also associated with salt intake. Transitive inference is a technique used in literature-based discovery (LBD) to synthesize new knowledge from disjointed knowledge domains [27]. If concepts A and C from separate knowledge domains are each associated with concept B, a novel association is inferred between A and C through B. This technique is useful for exploring new insights and directions for future research in a new area. 

**Figure 3:**
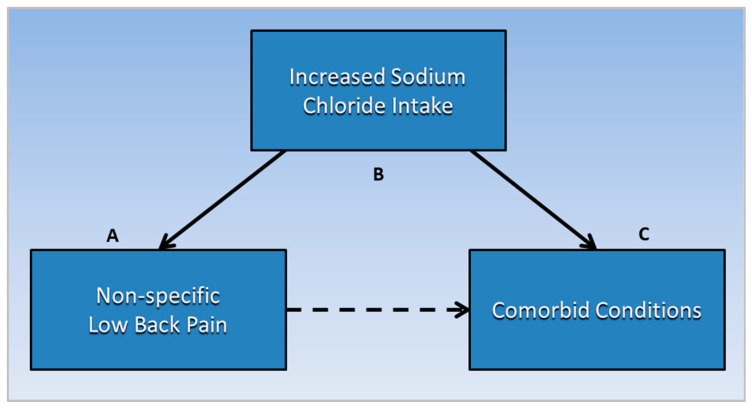
Non-specific low back pain (**A**) is transitively linked to comorbid conditions (**C**) through the common mediating factor of increased sodium chloride intake (**B**). Future studies should investigate prevalence of posterior lumbar subcutaneous edema in conditions comorbid with non-specific low back pain.

As shown in Figure 3, the following subsections describe evidence transitively linking non-specific LBP (A) with specific comorbid conditions (C), through the common mediating factor of increased salt intake (B). For evidence linking non-specific LBP with increased sodium chloride intake and PLSE, the reader is referred to Section 2 and Section 3 and Figure 2 of this paper. Future studies should investigate prevalence of PLSE in conditions comorbid with non-specific LBP.

### 4.1. Migraine Headache and Low Back Pain

Earlier research found that patients had a high prevalence of migraine headache following back pain [28,29]. More recently, a positive association of primary chronic headaches with persistent LBP was found in a systematic review of 14 studies [30], with odds ratios across a variety of populations ranging from 1.55 (95% CI 1.13–2.11) to 8.00 (95% CI 5.3–12.1). The researchers noted that “there might be a particular association between migraine and persistent back pain mediated through a specific biological mechanism.” Of relevance, clinical trials show lower salt intake is associated with reduced headaches [31,32], and migraine headache pain has been linked to withdrawal symptoms of salt intake [33], suggesting that salt mediates the association of headache with LBP. Importantly, increased water retention, similar to edema linked to salt intake, has been observed before the onset of migraine [34].

### 4.2. Hypertension and Low Back Pain

People with hypertension have a greater reduction in blood pressure from reduced sodium intake compared to people with normal blood pressure, and populations with lower sodium intake have overall lower blood pressure compared to other populations [35]. Low sodium intake in combination with the Dietary Approaches to Stop Hypertension (DASH) diet has been shown to reduce prehypertension or stage 1 hypertension [36].

Nevertheless, some studies found an inverse association of hypertension with LBP [37], which contradicts a potential relationship between high salt intake and non-specific LBP. However, the relationship between LBP and hypertension is complicated by the fact that chronic pain increases blood pressure through sympathetic nervous system responses [38]. This relationship between chronic pain and hypertension is reversed in acute pain in which sensitivity to pain is lowered through hypertension-associated hypoalgesia, which could account for inverse associations of acute LBP with hypertension in some studies. Furthermore, pain relief through use of nonsteroidal anti-inflammatory drugs and acetaminophen increases blood pressure by decreasing sodium excretion and increasing vascular volume, prompting a reminder to patients taking these drugs to “limit their sodium intake” [39]. Relieving LBP with such medications that increase hypertension could also account for inverse associations of LBP with hypertension.

### 4.3. Cardiovascular Disease and Chronic Low Back Pain

A study of Spanish twins found that chronic LBP was associated with a 2.69 odds ratio of lifetime myocardial infarction (95% CI 1.35–5.36), and a 2.58 odds ratio of other heart diseases over a lifetime (95% CI 1.69–3.93) [40]. Although a dose–response relationship between cardiovascular disease (CVD) and salt intake is unclear, a recent systematic review and dose–response meta-analysis found that each gram of sodium intake was associated with a 6% increased risk of CVD [41]. Moreover, a high-salt diet fed to rats was shown to increase damage to cardiomyocytes of the heart, independently of blood pressure [42]. This evidence infers that salt may transitively link chronic LBP with CVD.

### 4.4. Venous Thromboembolism and Severe Low Back Pain

Venous thromboembolism (VTE) is associated with severe LBP [43]. Researchers reviewed multiple case histories of patients with VTE and found that patients with inferior vena cava thrombosis frequently presented with initial symptoms of inexplicable lower back pain [44]. VTE is also related to sodium imbalance in hyponatremia and hypernatremia [45]. A high-salt diet increased plasma fibrinogen, platelet counts, and neutrophils in a mouse model [46]. Elevated serum sodium levels in a mouse model also increased secretion of von Willebrand factor (vWF) which initiates blood clots, and plasma vWF and stroke risk are positively associated with elevated serum sodium in the Atherosclerosis Risk in Communities Study [47].

### 4.5. Liver Disease and Back Pain

An early study found that back pain was the most common complaint among 54% of 239 patients with chronic hepatitis C [48]. Patients with liver cirrhosis had an increased incidence of spondylodiscitis of the spine with non-specific back pain [49]. Liver function in mice is harmed by excessive salt intake, which can induce fibrosis through excessive production of reactive oxygen species [50], and transcriptome sequencing technology demonstrated that a high-salt diet caused liver abnormalities in the metabolism of numerous substances and at least 15 enzymatic activities in mice [51].

### 4.6. Respiratory Disorders and Low Back Pain

A systematic review of 16 studies found a significant association between LBP and respiratory disorders, including respiratory infections, asthma, certain allergies, and dyspnea, but not chronic obstructive pulmonary disease [52]. The researchers suggested that biomechanical and immunological factors were among potential causes of the relationship of respiratory disorders with LBP, and further studies were recommended. A later prospective study that followed individuals from the Stockholm Public Health Cohort found that having asthma or chronic obstructive pulmonary disease at baseline was associated with a greater risk of LBP after four years follow up [53].

Of relevance, salt is a nutritional immunological factor associated with COVID-19 [54], a respiratory illness with SARS-CoV-2 infection that includes symptoms of shortness of breath, fever, congestion, headache, and body aches [55]. Changes in immune response from sodium chloride promote inflammation with increased inflammatory cytokines [56], which are prevalent in severe cases of COVID-19. The gummy yellow fluid observed to fill the air sacs of lungs in seriously ill COVID-19 patients appears similar to the yellow fluid in pulmonary edema [57,58], which could be related to excessive sodium intake. 

A meta-analysis of 51 studies investigated prevalence of musculoskeletal and neurological manifestations in COVID-19. Next to cases with myalgia and headache, with prevalence of 19% and 12%, respectively, general back pain was prevalent in 10% of COVID-19 cases [59]. Another study found that hospitalized COVID-19 patients complained of pain an average of 2.2 days before admission, and among 133 COVID-19 patients admitted with pain, LBP accounted for 43.6% of complaints [60].

### 4.7. Chronic Kidney Disease and Low Back Pain

Excessive salt intake harms kidneys by activating inflammatory responses and inducing fibrosis in renal tissue, which reduces kidney blood vessel density [22]. A low-sodium diet is useful in all stages of chronic kidney disease to reduce fluid overload, hypertension, and proteinuria in non-dialysis patients [61]. Moreover, a cross sectional study found that hemodialysis is associated with non-specific LBP in a majority of patients receiving hemodialysis, and LBP is associated with lower health-related quality of life among hemodialysis patients [62]. 

### 4.8. Pregnancy Complications and Low Back Pain

LBP affects 86% of pregnant women during the third trimester, and may persist for many months after delivery [63]. It is not clear if lordosis that normally occurs in the curvature of the lumbar spine during pregnancy is a causative factor of low back pain. Pregnant women with low back pain have worse quality of life and higher levels of sick leave than other pregnant women. Whether LBP in pregnancy and non-specific LBP “share similar underlying physiological mechanisms is still up for debate.” Furthermore, a previous history of LBP before pregnancy is one of the strongest predictors of LBP during pregnancy. Although earlier studies showed no benefit from sodium restriction in preventing pregnancy complications, a more recent study of Danish pregnant women found that lower dietary sodium intake to reduce risk of cardiovascular disease was associated with decreased risk of hypertensive disorders of pregnancy, including gestational hypertension and preeclampsia [64].

### 4.9. Multiple Sclerosis and Low Back Pain

A recent systematic review found that the prevalence of LBP in the French multiple sclerosis (MS) population ranged between 41.6% to 52.4%, which is two to three times as high as in the general population [65]. The study found that LBP in MS is the leading cause of limitation in daily activities, but the pathophysiology of LBP in MS remains unknown. Another study found that medium and high levels of dietary sodium compared to low levels consumed by MS patients were associated with 2.75 to 3.95 times worse clinical and MRI outcomes, respectively [66]. Excess dietary sodium chloride is proposed to induce pro-inflammatory mechanisms in the pathophysiology of MS [67], and high salt concentrations in experimental animal models produced pathological changes in the structure of myelin sheaths that protect neurons, which researchers suggested could explain demyelination in MS [68].

## 5. Conclusions

Novel primary prevention approaches to identify and mitigate the cause of non-specific LBP have potential to reduce disability worldwide. This paper presented evidence that posterior lumbar subcutaneous edema potentially mediates the association of increased dietary salt intake with non-specific low back pain. Salt is also a mediating factor in the transitive relationship between LBP and many comorbid conditions. More research is needed to confirm the mediating role of posterior lumbar subcutaneous edema in the proposed association of increased sodium chloride intake with non-specific low back pain and comorbid conditions. Additionally, future clinical research should explore the feasibility of using a salt-reduced diet for the reduction and prevention of non-specific LBP.
